# Efficacy of Different Doses of Multiple Micronutrient Powder on Haemoglobin Concentration in Children Aged 6–59 Months in Arusha District

**DOI:** 10.1155/2019/8979456

**Published:** 2019-02-03

**Authors:** Dyness Kejo, Pammla Petrucka, Haikael Martin, Theobald C. E. Mosha, Martin E. Kimanya

**Affiliations:** ^1^Nelson Mandela African Institution of Science and Technology (NM-AIST), Department of Food Science and Nutrition, P.O. Box 447, Arusha, Tanzania; ^2^Tanzania Agriculture Research Institute (TARI), P.O. Box 1253, Tengeru, Arusha, Tanzania; ^3^University of Saskatchewan, College of Nursing, Saskatoon, Canada; ^4^Adjunct, NM-AIST, Arusha, Tanzania; ^5^Sokoine University of Agriculture, Department of Food Technology, Nutrition and Consumer Sciences, P.O. Box 3109, Morogoro, Tanzania

## Abstract

In Tanzania's Arusha District, anaemia is a significant public health problem. Recently, home fortification with multiple micronutrient powder was recommended, and daily use of one sachet has shown to be effective. However, it is a challenge for deprived families with low income to afford the daily sachet. The aim of this study was to compare the efficacy of different administration frequencies of micronutrient powder in reducing anaemia in children aged 6–59 months. This research used a community-based, randomized longitudinal trial design with the intent to treat anaemia. Children aged 6 to 59 months (*n*=369) were randomly assigned to one of four intervention groups which received, on a weekly basis, either five sachets (*n*=60), three sachets (*n*=80), two sachets (*n*=105), or one sachet (*n*=124) for six months; 310 children completed the study. Using the HemoCue technique, a finger-prick blood was taken at baseline, middle, and end points of the intervention to determine haemoglobin levels. The effect of treatment on haemoglobin was assessed with analysis of covariates with Bonferroni post hoc to test group difference (*p* > 0.05) from each other. At the end, haemoglobin levels were significantly higher in participants who received three or five sachets of micronutrient powder per week compared to those who received one or two micronutrient powder sachets per week (*p* < 0.05). The prevalence of illnesses was reduced from 65% to 30.5% in all groups. This finding indicates that economically challenged families may opt for three times per week sachet administration rather than a more costly daily administration. This trial is registered with PACTR201607001693286.

## 1. Introduction

Low haemoglobin concentrations, a measure of anaemia, are a significant global challenge not only at the individual level (i.e., development and illnesses) but also at the societal level (i.e., economic and social capital) [1]. Anaemia as an indicator of micronutrient deficiencies is mainly attributed to iron deficiency but also may be associated with vitamin A, folic acid, and vitamin B12 deficiencies and other non-nutritional causes like infection, loss of blood, and genetic disorder [2]. Infants and children are most vulnerable to micronutrient deficiency, given the high vitamin and mineral intake needed to support their rapid growth and adequate development. About 273 million children under the age of five years are affected by anaemia with the majority being located within developing countries [1, 3]. In Tanzania, more than half (57%) of the children under five years of age are anaemic [4].

Beyond the “anaemia level,” iron deficiency anaemia is the most common cause of anaemia in most parts of the world [5]. Generally, high-quality and fortified foods are unavailable and/or unaffordable for low-income families. A diversified diet and food fortification of staple foods are considered as the optimal approaches for minimizing micronutrient deficiencies, especially iron deficiency anaemia. Historically, achieving dietary diversity and widespread utilization of fortified food is a relatively slow process, for example, utilization of iodized salt has shown extremely slow uptake in Tanzania [6]. Use of fortified complementary foods (CFs) for young children is also a limited option in rural communities as fortified foods are simply not available but also eaten in such small quantities that are not sufficient to assume micronutrient intake from commercially fortified foods among children aged 6–59 months.

Home fortification with multiple micronutrient powder (MNP) is considered an opportunity when the CFs do not provide enough essential nutrients [7]. Micronutrient intervention with MNP is recognized as a cost-effective approach for reducing micronutrient deficiencies in low-income countries [3]. Micronutrient powder comes in sachets containing dry powder with micronutrients that can be added to any semisolid or solid food that is ready for consumption [8]. In most developing countries, such as Tanzania, cereal gruels are commonly used for complementary feeding. These are normally rich in phytate and tannins while concomitantly being deficient in protein and micronutrients, thus predisposing children to iron deficiency anaemia [3, 9–11].

Initially, MNP was developed to provide iron and other nutrients required for treating nutritional anaemia [8]. Many efficacy trials of MNP interventions to improve micronutrients status, especially iron status and anaemia, have been well established and ongoing [12–15]. The Government of Tanzania, in collaboration with development partners, has been accelerating efforts to alleviate micronutrient deficiencies in the country. Mothers are required to buy the MNP sachets daily from selected agents or a health facility. Research has shown that daily MNP supplementation produced the best result in terms of anaemia reduction [6, 16]; however, for poor families (most have more than one child under five years of age), it is expensive to buy one sachet at Tanzanian shillings 150 (approximately $0.08) on a daily basis. Therefore, this study assesses the efficacy of home fortification with MNP at a different dosage in relation to their affordability among affected children below five years in Arusha District.

## 2. Methods

### 2.1. Study Area

This study was done in Arusha District, Northern Tanzania. The District is primarily rural with a socioeconomic pattern similar to most rural districts in Tanzania, demonstrates cultural diversity, and reports childhood anaemia higher (84%) than previously reported District average prevalence (58%) [17]. Arusha District has twenty wards with at least one reproductive child health clinic for mothers and children in each ward. Three wards—Oldonyosambu, Oturumeti, and Seliani which serve 170, 182, and 200 children under five years, respectively—were randomly selected for inclusion in this study.

### 2.2. Study Design, Sample Size, and Randomization

The study was a community-based, randomized longitudinal trial with intent to treat anaemia. The intervention study was designed to last for six months with data collection at baseline, midline, and end line of the intervention. The sample size was determined using the statistical power analysis formula *n*=*z*^2^*p*(1 − *p*)/*d*^2^ [18] for anaemia assessment, where *n* = sample size, *p* = prevalence of anaemia (60%) [9], *z* = *z* value at 95% confidence (1.96), and *d* = level of significance (5%), with an anticipated attrition of 18% to follow-up yielded a total of 436 mother-child pairs. The purpose of the study and the benefits of consuming the multiple micronutrient supplements that contained iron (10 mg) were explained to the child mothers. Eligibility criteria were as follows: the family had to reside in the selected ward; the child must be 6–59 months of age, haemoglobin (Hb) levels from 7.0 to 9.9 g/dL, and children who started complementary foods. Exclusion criteria were as follows: Hb concentrations ≤6.99 g/dL or ≥10.0 g/dL, child who does not consume solid foods, chronic conditions such as type 1 diabetes mellitus, some inborn errors of metabolism, HIV infection, or known hasemoglobinopathies.

After the baseline assessment, eligible (*n*=369) children were randomly allocated (using a computer-generated list) to one of the four intervention groups. Group one (*n*=60) was given MNP five per week (5/wk); group two (*n*=80) was given MNP three times per week (3/wk); group three (*n*=105) was given MNP twice per week (2/wk); and group 4 (*n*=124) was given MNP once per week (1/wk). For this efficacy trial, the primary outcome was anaemia status. To control for confounders, all children aged 24 months and older were given a single dose of albendazole™ for deworming during the baseline assessment. Six to 11 months old children were not treated for helminthes.

### 2.3. Sampling Frame

The sampling frame was established, and 20 wards with health facilities offering Reproductive and Child Health (RCH) clinic services were identified and coded from 01 to 20. Three wards out of 20 wards were randomly selected using a table of random numbers (due to lack of funds few wards were selected). From these wards, all households with infants aged 6–59 months were identified and selected randomly with the help of village health workers with those who agreed to participate being invited to the RCH (as a meeting point) for baseline assessment. The study objectives were explained by the researcher to mothers/caregivers, and a written informed consent was obtained.

### 2.4. Intervention and Compliance Monitoring

Mothers/caregivers were instructed to mix the content of a full sachet of MNP with the child's prepared (cooked) complementary food by sprinkling contents as per sachet instructions. They were encouraged to mix the MNP when the food is warm and to ensure the child consumes all the food after addition of MNP. The MNP contained 15 vitamins and minerals. The nutrient content of 1 g of MNP was vitamin A (RE 400 *μ*g), vitamin D (5 *μ*g), vitamin E (5 mg), vitamin B1, B2, and B6 each (0.5 mg), folic acid (0.15 mg), niacin (6 mg), vitamin B12 (0.9 *μ*g), vitamin C (30 mg), iron (10 mg), zinc (4.1 mg), selenium (17 *μ*g), copper (0.56 mg), and iodine (90 *μ*g). The MNP was supplied in a single-dose sachet (1 dose = 1 sachet), and one pack contained 30 × 1 g sachets, based on the Recommended Nutrient Intake (RNI) for children [8].

In this study, community health workers were involved in the follow-up of study participants. They visited homes of participant children on a weekly basis to record supplement use and collected empty sachets weekly to monitor compliance. Their visits ensured the supplement was given to the targeted child and encouraged continued use of the supplement.

### 2.5. Data Collection

At baseline, the information on children and their families was collected through questionnaire-guided interviews (face to face) with the child's mother or caregiver. Data on basic demographics, breastfeeding, CF practices, and recent illnesses in the preceding 14 days were collected. During the intervention, community health workers recorded information about supplement acceptance, side effects, and health information including diarrhea, fever, cough, and other sickness and also behavior changes such as increase in appetite and increase in the level of physical.

### 2.6. Assessment of Haemoglobin Concentration

At baseline, midline, and end line, haemoglobin (Hb) concentration was measured by the HemoCue® Hb 201+ photometer (AB, Sweden) by qualified technicians. Safety lancets were used to obtain the finger-prick blood which was collected in the microcuvettes. Alcohol swabs were used to clean the fingers before pricking. The first drop of blood was wiped out by cotton wool, while the second drop was collected in the microcuvette. Blood sample in the microcuvette was loaded in the calibrated HemoCue® photometer, and Hb concentration read to the nearest 0.1 g/dL. Categorization of Hb levels was done based on the WHO standard, where children with Hb level <11 g/dL were considered anaemic: mild (10–10.9 g/dL), moderate (7–9.9 g/dL), and severe (<7 g/dL) anaemia [7]. HemoCue machine was calibrated using an available machine at RCH clinic.

### 2.7. Ethical Considerations

The study received ethical clearance from the Ethics Committee of the National Institute for Medical Research (NIMR) Tanzania. Written informed consent was obtained from the mothers/caregivers at the time of enrolment in the study.

### 2.8. Data Analysis

The data collected were entered, cleaned, coded, and analyzed using SPSS™ Version 20. Analysis was performed on the intension to treat basis. Descriptive statistics were calculated and reported as means and standard deviation. Analysis of variance (ANOVA) was done on continuous variables with normal distributions to test whether there were significant group differences (*F*-test). The effect of treatment on haemoglobin was assessed with analysis of covariates including treatment as a fixed factor and sex and age as covariates. Significance of Boniferon post hoc test was used to specifically test whether each of the 4 supplementation groups differed significantly (*p* < 0.05) from each other. Differences in categorical variables were assessed using chi-squared tests.

## 3. Results

**Figure 1:**
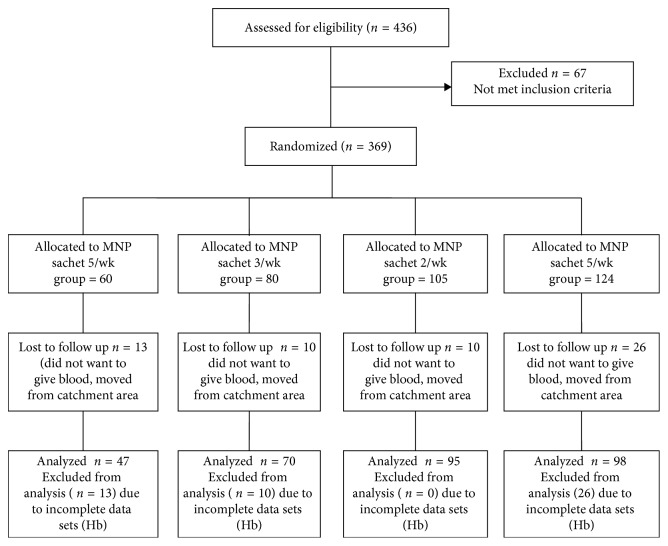
Flow diagram of subject's progress through the study.

After baseline assessment, 369 children met inclusion criteria and enrolled in the study; 16% (*n*=59) were lost to follow-up and could not be assessed at the end of the intervention.  Figure 1 shows the flow diagram according to CONSORT guideline. The compliance at the end of the intervention was 84%.

### 3.1. Baseline Characteristics

Gender distribution among participating children was 58% (*n*=180) males and 42% (*n*=130) females. The randomized four intervention groups did not differ significantly in baseline characteristics, except for a significant age difference (*p* < 0.001) between groups (Table 1).

Table 2 shows change in Hb concentration during the intervention. There was an increased Hb concentration in all the intervention groups (Table 2). The average Hb concentration increased from 9.0 ± 0.70 g/dL at baseline to 11.32 ± 0.52 g/dL at the end line of study in children consuming five sachets per week compared to others. Hb levels increased more in groups consuming three or five sachets per week compared to groups consuming one or two sachets per week. After six months of the intervention, there was no significant (*p* ≥ 0.05) difference in the increase in Hb concentration among those consuming MNP three or five times per week.

Table 3 shows the mean difference in increase of Hb concentration for the six months of the intervention. At the end of intervention, groups consuming 5/wk, 3/wk, and 2/wk Hb concentration were increased by 1.7 g/dL, 1.5 g/dL, and 1.2 g/dL, respectively. No significant difference (*p* ≥ 0.05) was found between children consuming five and three sachets per week and children consuming two sachets per week versus three sachets per week.

### 3.2. Prevalence of Anaemia

At the end of the intervention, about 52%, 67%, and 79% of the children in the groups who received 2/wk, 3/wk, and 5/wk sachets, respectively, had moved from being anaemic to having normal Hb levels >11 g/dL (Table 4). This suggests that these intervention groups were effective in decreasing prevalence of anaemia.

At baseline, about 65.0% (*n*=200) of the children were having diarrhea, fever, cough, and others having more than one type of diseases. It was observed that after taking MNP for 6 months, children were significantly (*p* < 0.05) better. At the end of intervention, percentage of children with symptoms of illness decreased from 65% to 30.6% (Table 5). Hence, the introduction of MNPs appears to have contributed to the reduction of the incidence of infectious diseases in supplement recipients.

## 4. Discussion

In this study, more than half of the subjects were anaemic (displaying low Hb < 11 g/dL). The high (84%) prevalence of anaemia exceeded reported regional (58%) and national prevalence (57%); hence, the potential to introduce an intervention was deemed meritorious. According to the WHO [7], in populations, where the prevalence of anaemia in children under 5 years is 20% or higher, point-of-use fortification of CFs with iron-containing MNP among young children is recommended to improve the iron status and reduce anaemia.

In the study communities, dietary factors, such as poor intake of iron-rich foods, were identified as a contributing cause of anaemia [17] as the most frequently consumed diets are predominantly plant-based with low iron content and have antinutritional factors (e.g., phytate) which hinder iron absorption. In settings, such as Arusha District, where iron-rich foods are lacking, iron supplementation is needed for vulnerable groups, including young children. Such efforts often include point-of-use fortification with MNP supplementation as part of an infant and young child-feeding strategy to improve nutrient intake via CFs by children from six months of age and above [3]. In this study, MNP dosages of five sachets or three sachets per week were found to be effective in improving Hb concentrations as well as addressing mild and moderate anaemia. The significant effect of MNP in raising plasma Hb concentration was evidenced in the 5/wk and 3/wk sachets intervention groups (1.7 g/dL and 1.5 g/dL respectively) when compared with the 2/wk (1.2 g/dL) recipient group. The 3/wk and 5/wk sachets weekly administration of MNP was significantly more effective than lesser frequencies.

Through this study, MNP supplementation was demonstrated to be effective in reducing the prevalence of anaemia to 43% (Table 4). Consequently, children receiving MNP supplementation of greater than two sachets per week were more likely to build up sufficient iron storage to mitigate or avert anaemia recurrence for the next 6 months. This finding is in line with other studies on the effectiveness of home fortification [16, 19–22]. Results from this study showed that MNP was effective in reducing the prevalence of anaemia and improving Hb concentration in both groups (three and five sachets per week), again mirroring the results of previous studies in several developing countries [15, 23–25]. In this study, there is no significant increase in Hb concentration in either twice or thrice per week supplementation groups similar with 3/wk and 5/wk. Children recovered from anaemia differently according to MNP dose given, indicating iron absorption was more effective in accordance with the dosages. Again, this finding aligns with previous micronutrient supplementation trials which suggest DMM as the optimal treatment to improve both anaemia and Hb concentration by more than 30% of the studied children [6, 11, 16].

The effectiveness of MNP in reducing the prevalence of anaemia in pediatric populations with high rates of mild and severe anaemia was confirmed in a Peruvian clinical trial evaluation [12, 26]. The current study went further to show similar effectiveness in anaemia reduction in 3/wk as that in 5/wk. Although reduction of anaemia was also observed in the twice per week supplementation group, the reduction was relatively smaller (52%) than that in the five (79%) and three (67%) per week supplementation groups. At baseline, all participating children were moderate anaemic with Hb level below 10 g/dL, and during the end line assessment, most had an improved Hb level.

During the intervention period, mothers were prompted to recount any observed behavior changes in their children after supplementation. Some reported positive changes in physical activities (i.e., engaged more in play activities) and appetite. The positive health effect of MNP observed by all families included improved immunity as reflected in a reduction in the frequency of illness among the participating children. Reported morbidity among participating children was reduced significantly from baseline prevalence of 65% to terminal prevalence of 30.6%. Changes in stool color were observed in children when consuming MNP, but the parents were assured that this alteration was not a concern or adverse effect. Point-of-use fortification improved the micronutrient status of recipient children, which has led to improved child health outcomes including reducing morbidity and improving appetite, which may, in the future, lead to improved future growth and social capacities. We acknowledge some limitations on study design, i.e., age difference among treatment groups. Seasonality and change of dietary intake might influence the increase of haemoglobin during intervention, which was not included during analysis.

## 5. Conclusion

This study, conducted on children with high anaemia prevalence at baseline, provides evidence that home fortification with MNP increases Hb concentration and reduces anaemia in an administration frequency-dependent manner. The administration of MNP supplementation three or five times per week produced optimal results in terms of anaemia reduction potentiating an improvement of micronutrient status and also can improve child health outcomes, including reduced morbidity, as well as increased appetite and other functional outcomes. Strategically, home fortification with MNP beginning at 6 months would give additional public health benefit by promoting the timely introduction of CFs and enhancing the likelihood of adequate micronutrient access. The study strongly suggests that low-income families can use a less expensive approach using three sachets rather than a daily administration while gleaning fairly similar results and benefits to their children. This evidence is both relevant and timely, given the current Tanzania and global context in which “private-public partnerships” are increasingly advancing primary healthcare initiatives such as home fortification with MNP by selling and creating awareness to mothers/caregivers to self-secure the MNP and assume coresponsibility for reduction of childhood anaemia. More studies are necessary to verify the optimal dose and economic feasibility of using MNP in a range of contexts and target subpopulation.

## Figures and Tables

**Table 1:** Baseline characteristics of the study population.

Characteristics	Treatment group				
	5/wk (*n*=47)	3/wk (*n*=70)	2/wk (*n*=95)	1/wk (*n*=98)	*p* value^*∗*^
Child age (months)	17.7 ± 7.9	18.5 ± 6.9	20.1 ± 10.1	14.4 ± 7.9	<0.001
Birth weight (kg)	3.1 ± 0.5 (32)	3.4 ± 0.5 (43)	4.2 ± 11.5 (70)	2.9 ± 0.5 (66)	0.6
Mother's age (years)	25.3 ± 4.3	25.7 ± 4.4	25.8 ± 5.4	25.9 ± 4.7	0.1
Sex, male (%)	44.7	64.3	64.2	54.1	0.08
EBF (%)	14.9	12.9	20.1	9.2	0.1
Stunting (%)	53.2	44.3	50.5	46.9	0.8
Underweight (%)	21.4	24.3	27.4	21.4	0.8
Wasting (%)	14.9	12.9	16.8	9.2	0.1
Marital status, married (%)	87.2	90.0	91.6	92.9	0.7

Values are means ± SD; ^*∗*^significant at the 0.05 level.

**Table 2:** Change in haemoglobin concentrations (mean ± SD) during intervention.

Duration	Treatment groups				
	5/wk	3/wk	2/wk	1/wk	*p* value^*∗*^
Baseline	9.0 ± 0.70	9.2 ± 0.78	9.0 ± 0.79	9.0 ± 0.67	0.5
Midline	10.02 ± 0.60	9.8 ± 0.78	9.6 ± 0.90	9.3 ± 0.71	0.00
End line	11.32 ± 0.52	11.10 ± 0.8	10.80 ± 1.02	9.60 ± 0.70	0.00

^*∗*^Based on ANOVA (continuous variable).

**Table 3:** Mean difference of haemoglobin concentration between intervention groups.

Duration	Comparison group		Mean difference (a − b) ± SE	*p* value^b^	95% CI
	(Dose (a))	(Dose (b))			
Midline	1/wk	2/wk	−0.45 ± 0.12^*∗*^	0.001	−0.76–−0.15
		3/wk	−0.56 ± 0.12^*∗*^	0.00	−0.89–0.24
		5/wk	−0.77 ± 0.14^*∗*^	0.00	−1.14–0.41
	2/wk	1/wk	0.45 ± 0.12^*∗*^	0.00	0.47–0.76
		3/wk	−0.11 ± 0.12	1.00	−0.43–0.21
		5/wk	−0.21 ± 0.14	0.01	−0.68–0.04
	3/wk	1/wk	0.56 ± 0.12^*∗*^	0.00	0.24–0.88
		2/wk	0.11 ± 0.12	1.00	−0.21–0.43
		5/wk	−0.21 ± 0.15	0.88	−0.60–0.18
	5/wk	1/wk	0.77 ± 0.14^*∗*^	0.00	0.41–1.14
		2/wk	0.320 ± 0.14	1.00	−0.05–0.69
		3/wk	0.212 ± 0.15	0.88	−0.18–6.00

End line	1/wk	2/wk	−1.21 ± 0.12^*∗*^	0.00	−1.54–0.88
		3/wk	−1.51 ± 0.13^*∗*^	0.00	−1.85–−1.16
		5/wk	−1.69 ± 0.15^*∗*^	0.00	−2.09–−1.31
	2/wk	1/wk	1.21 ± 0.12^*∗*^	0.00	0.88–1.54
		3/wk	−0.29 ± 0.29	0.13	−0.64–0.05
		5/wk	−0.49 ± 0.15^*∗*^	0.006	−0.88–−0.99
	3/wk	1/wk	1.51 ± 0.13^*∗*^	0.00	1.16–1.85
		2/wk	0.29 ± 0.13	0.13	−0.05–0.64
		5/wk	−0.19 ± 0.15	1.00	−0.60–0.22
	5/wk	1/wk	1.69 ± 0.15^*∗*^	0.00	1.31–2.09
		2/wk	0.49 ± 0.15^*∗*^	0.00	0.09–0.88
		3/wk	0.19 ± 0.15	1.00	−0.22–0.6

^*∗*^The mean difference is significant at 0.05 levels. ^b^Adjusted for multiple comparisons: Bonferroni

**Table 4:** The effect of micronutrient powders supplementation on prevalence of anaemia.

Variable	Treatment groups				
	1/wk	2/wk	3/wk	5/wk	Total
Normal	0	51.6	67.1	78.7	42.9
Mild	38.8	28.4	24.3	19.1	29.4
Moderate	61.2	20.0	8.6	2.1	27.7

**Table 5:** Impact of supplementation on morbidity.

Morbidity	No. of respondents	%
Illness at baseline	200	65.0
Illness after intervention	95	30.6

## Data Availability

The data used to support the findings of this study are available from the corresponding author upon request.
